# Differential p53 protein expression in breast cancer fine needle aspirates: the potential for in vivo monitoring

**DOI:** 10.1054/bjoc.2001.2064

**Published:** 2001-10

**Authors:** H M-L Ball, T R Hupp, D Ziyaie, C A Purdie, N M Kernohan, A M Thompson

**Affiliations:** Department of Surgery and Molecular Oncology, Department of Molecular and Cellular Pathology, Ninewells Hospital, Dundee, DD1 9SY, UK

**Keywords:** FNA (fine needle aspiration), breast cancer, p53, Western blotting

## Abstract

Fine needle aspiration (FNA) biopsy is the least invasive method of sampling breast cancer in vivo and provides material for breast cancer diagnosis. FNA has also been used to examine cellular markers to predict and monitor the effects of therapy. The aim of this study was to assess the accuracy of using FNA material compared with resected cancer for Western blotting studies of the p53 pathway, a key to tumour response to radiotherapy and chemotherapy. Paired samples of breast cancer FNAs collected pre-operatively and post-operatively were compared with tissue samples obtained at the time of surgical resection. Western blots were probed for p53 using the antibodies DO12 and DO1, and for levels of downstream proteins p21/WAF1 and p27. The protein extracted by FNA was sufficient for up to 5 Western blot studies. p53 expression and phosphorylation did not differ significantly pre- and post-operatively, indicating that intra-operative manipulation does not affect p53 expression or downstream activation in breast cancer. However, expression of p53, p21 and p27 varied between individual patients suggesting a range of p53 pathway activation in breast cancer. Immunohistochemistry confirmed that the cancer cells accounted for the protein expression detected on Western blots. FNA yields adequate protein for Western blotting studies and could be used as a method to monitor p53 activity in vivo before and during anti-cancer treatment possibly providing early evidence of tumour response to therapy. © 2001 Cancer Research Campaign  http://www.bjcancer.com


*British Journal of Cancer* (2001) **85**(8), 1102–1105
© 2001 Cancer Research Campaign
doi: 10.1054/ bjoc.2001.2064, available online at http://www.idealibrary.com on IDEAL
http://www.bjcancer.com

### Differential p53 protein expression in breast cancer fine needle aspirates: the potential for in vivo monitoring

**HM-L Ball[1], TR Hupp[2], D Ziyaie[1], CA Purdie[2], NM Kernohan[2] and AM Thompson[1]**

[1]Department of Surgery and Molecular Oncology & [2]Department of Molecular and Cellular Pathology, Ninewells Hospital, Dundee DD1 9SY, UK

**Summary** Fine needle aspiration (FNA) biopsy is the least invasive method of sampling breast cancer in vivo and provides material for breast cancer diagnosis. FNA has also been used to examine cellular markers to predict and monitor the effects of therapy. The aim of this study was to assess the accuracy of using FNA material compared with resected cancer for Western blotting studies of the p53 pathway, a key to tumour response to radiotherapy and chemotherapy. Paired samples of breast cancer FNAs collected pre-operatively and post-operatively were compared with tissue samples obtained at the time of surgical resection. Western blots were probed for p53 using the antibodies DO12 and DO1, and for levels of downstream proteins p21/WAF1 and p27. The protein extracted by FNA was sufficient for up to 5 Western blot studies. p53 expression and phosphorylation did not differ significantly pre- and post-operatively, indicating that intra-operative manipulation does not affect p53 expression or downstream activation in breast cancer. However, expression of p53, p21 and p27 varied between individual patients suggesting a range of p53 pathway activation in breast cancer. Immunohistochemistry confirmed that the cancer cells accounted for the protein expression detected on Western blots. FNA yields adequate protein for Western blotting studies and could be used as a method to monitor p53 activity in vivo before and during anti-cancer treatment possibly providing early evidence of tumour response to therapy. © 2001 Cancer Research Campaign http://www.bjcancer.com

**Keywords**: FNA (fine needle aspiration); breast cancer; p53; Western blotting

The *p53* gene is central to the cellular response to genotoxic damage (Kastan et al, 1991) and chemotherapeutic agents (Linke et al, 1996). A coordinated downstream response from p53 involves activation of a number of target genes containing p53-binding sites, such as mdm-2 and p21/WAF1 (Bueso-Ramos et al, 1996; Steele et al, 1998; Jones et al, 1999).

Mutation or overexpression of p53 protein has been observed in up to 52% of primary breast cancers (Ziyaie et al, 2000). Unlike most other epithelial malignancies, in breast cancer abnormalities in p53 protein *function* rather than specific p53 mutations are more common (Andersen and Borresen, 1995; Ziyaie et al, 2000). p53 status is an important determinant of tumour response to anti-neoplastic agents (Lowe et al, 1994), with specific mutations of p53 associated with poor response to systemic therapy (Elledge et al, 1995; Aas et al, 1996).

Absolute levels of p53 can be identified using the monoclonal antibody DO12, which recognises a cryptic epitope in the core domain of p53 (Vojtesek et al, 1995). DO1, an anti-p53 antibody, recognises amino acids 20–26 at the n terminus of p53 and is commonly used for immunohistochemical staining for p53 (Stephen et al, 1995).

As a consequence of p53 activation there is increased expression of effector genes including *mdm2*, *p21/WAF1* and *p27*. Whilst the mdm-2 protein controls the biological activity of p53 and targets it for destruction (Haupt et al, 1997), both p21/WAF1 and p27 act as cyclin-dependent kinase (cdk) inhibitors, preventing phosphorylation of Rb and release of E2F thus interfering with S-phase signalling (Sherr and Roberts, 1999). High expression of p21/WAF1 may be related to short relapse-free survival in breast cancer (Barbareschi et al, 1996) although the evidence is conflicting (Elledge and Albred, 1998), and p21/WAF1 mutations are not common in cancers (Shiohara et al, 1997). p27 is rarely mutated in human primary breast carcinomas and breast cancer cell lines (Sgambato et al, 1997); however high levels have been found in many cell lines even during exponential growth phase, despite its known role as an inhibitor of cell cycle progression (Sgambato et al, 1997).

If it were possible to examine the p53 pathway in vivo in patients, and thus predict the individual tumour response to chemotherapy, patients likely to respond to chemotherapy could proceed with subsequent courses of treatment while others were spared further, ineffective, treatment and directed to alternative therapy. Fine needle aspiration (FNA) is used as a routine diagnostic investigation, furthermore FNA samples obtain representative tumour cell populations from a breast cancer (Brotherick et al, 1995) causing minimal tumour disruption and are both acceptable to patients and yield good material for study. FNAs have been found to be a viable alternative to intraoperative conventional surgical biopsy for biochemical analysis of prognostic factors (Kuner et al, 2000). Cancer cells extracted by FNA have been used for cytology (Pelosi et al, 1994), polymerase chain reaction (Gudlaugsdottir et al, 2000), DNA sequencing analysis (Lavarino et al, 1998), fluorescence in situ hybridisation analysis (Rao et al, 1998) and flow cytometry (Makris et al, 1997). In addition, FNA reliably allows immunocytochemical determination of biological variables in primary breast carcinoma (Nizzoli et al, 2000). The functional significance of key genes in a given cancer may be most accurately determined by detecting the protein. The aims of this study were to assess the feasibility of using fine needle aspiration to examine protein expression of genes from the p53 pathway in

*Received 10 April 2001*
*Revised 9 July 2001*
*Accepted 13 July 2001*

*Correspondence to:* AM Thompson breast cancer in vivo at multiple time points and to confirm that the cancer tissue (rather than the normal tissues) accounted for the proteins detected by Western blotting.

### MATERIALS AND METHODS

### Fine needle aspiration and human tissue

Fine needle aspirates (FNA) were obtained by a single operator with a 21G needle and 10 ml syringe, passing the needle through the cancer 10 times with application of 1–2 ml suction (Hartley et al, 1988). 500 µl of chilled phosphate-buffered saline (PBS) was then drawn up, the needle contents expelled into a chilled eppendorf on ice then spun at 5000 ***g*** (4˚C) for 5 minutes. The supernatant was aspirated and the pellets snap frozen in liquid nitrogen stored at –70˚C.

FNAs were obtained from 16 consenting patients prior to mastectomy or wide local excision for primary untreated breast cancer and immediately post-operatively from the resected specimen adjacent to blocks used for histological assessment. Post-operative samples were collected no later than 15 minutes following excision. Within 15 minutes of excision, 3 $mm^2$ tumour biopsies were also excised and snap frozen in liquid nitrogen, then stored with the corresponding aspirates at –70˚C. Local Ethical Committee approval was obtained.

### Protein extraction and analysis

Protein was extracted using urea lysis buffer (8.7 ml urea (8 M), 1 ml dithiothreitol (1 M), 50 µl Triton (10%), 50 µl NaCl (5 M), 200 µl Hepes (1 M, pH 7.6). Tumour aspirate pellets, Hela cell pellets (as a positive control for p53, p21 and p27) and snap frozen tumour were homogenised in three times the volume of urea lysis buffer. Samples were left on ice for 15 minutes then spun at 13 000 ***g*** (0˚C) for 10 minutes. The supernatant was transferred into prechilled eppendorfs, snap frozen and stored at –70˚C. Protein concentration was determined at 595 λ (Bradford, 1976). Bradford assay showed a 3-fold dilution of protein when the barrel of the syringe was washed through with PBS for the second time. To maximise yield, both washes were combined before being spun down.

The protein extracted from the FNAs and tumour tissue was used to perform repeat gels and Western blots of the samples for confirmation of expression profile comparing pre-operative FNA vs. post-operative FNA vs. pathology block in all 16 patients.

### SDS/PAGE gel electrophoresis

SDS/PAGE was carried out using both 10% and 15% polyacrylamide resolving gels and standard Laemlli buffer. The 16 tumour lysates were run on a 10% polyacrylamide gel, before being probed with the appropriate antibody. Arn8 Lysates with χ radiation and DRB were used as the positive control (Blaydes et al, 2000).

### Antibodies

Anti-p53 antibodies DO-1 (p53 n-terminus specific) and DO-12 (for absolute levels of p53) supplied by Moravian Biotechnology, Czech Republic, have been described previously (Blaydes and Hupp, 1998; Blaydes et al, 2000). p21 WAF1 (Ab1) was supplied by Oncogene Research Products, Calbiochem and p27 (C-19) antibody from Santacruz Biochemistry. Immunoblotting was carried out using standard methodology. Blots were examined and interpreted independently by 3 of the authors (TRH, AMT, HMB); and there was unanimity for each antibody for all samples.

### Immunohistochemistry

Immunohistochemistry was performed on 10 neutral-buffered formalin-fixed, paraffin-embedded sections taken from tissue adjacent to that used for Western blotting. Sections were microwaved for 15 minutes in citric acid (0.1 M, pH 6.0) for antigen retrieval and probed with monoclonal antibodies DO1, WAF1 or p27. A horseradish peroxidase (HRP) detection system using diaminobenzidine (DAB) substrate and cells were counterstained with haematoxylin. Coverslips were mounted using organic mountant (DPX). Slides were examined independently by two of the authors (CP, NK) blinded to the Western blotting data. There was unanimity in the scoring. Immunohistochemical data were cross-tabulated with the Western blotting results.

### RESULTS

Protein was extracted successfully from all FNA and cancer samples. Fine needle aspiration yielded up to 350 µg of protein for Western blots.

There was no difference in p53 expression between FNA samples taken pre-operatively and post-operatively on Western blotting (Figure 1; tumour samples 13a and 13b). However, an additional (third) band was observed in the homogenised cancer specimen 13c not seen in 13a or 13b (the FNAs) for one cancer. Matched venous lymphocyte protein from the same patient did not demonstrate any p53 expression with DO12 antibody. For DO1, p21/WAF1 and p27 expression was consistent for the FNAs and tumour sample both in terms of level of expression and species detected.

Individual cancers showed different p53 levels when probed with DO12 (Figure 2A), with absolute levels of p53 (DO12) in 12/16 patients and p53 expression using an antibody for the N-terminus (DO1) identified in 10/16 patients. 8 of the 16 tumours had detectable p21 (Figure 2B), but only 2 cancers had detectable p27 (Figure 2C). In 2 patients no DO12, DO1, p21/WAF1 or p27 was detected on Western blotting despite positive controls.

Repeat gels, Western blots and probing demonstrated consistent expression/absence of expression for each sample and every protein species.

### Immunohistochemistry

Sections of the 16 tumours stained by immunohistochemistry (IHC) confirmed cancer cells were responsible for the positive bands on Western blotting. In every case IHC-staining intensity correlated with the Western blotting data (Figure 3).

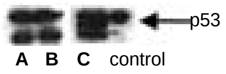

**Figure 1** Western blot of patient 13 and positive control (Hela cells) from (**A**) preoperative FNA, (**B**) post-operative FNA and (**C**) excised tumour, demonstrating principal p53 species (arrowed) and additional, smaller species in the FNA and excised tumour samples

*© 2001 Cancer Research Campaign* 

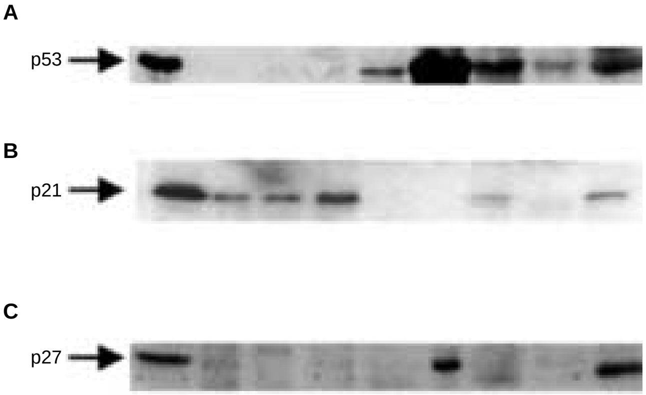

**Figure 2** Western blots of cancers 1 to 8 (equal total protein in each lane) and positive control (Hela cells) demonstrating expression of (**A**) p53 using DO12, (**B**) p21/WAF1 using Ab1 and (**C**) p27 using C-19; note levels of expression vary greatly between individual patients

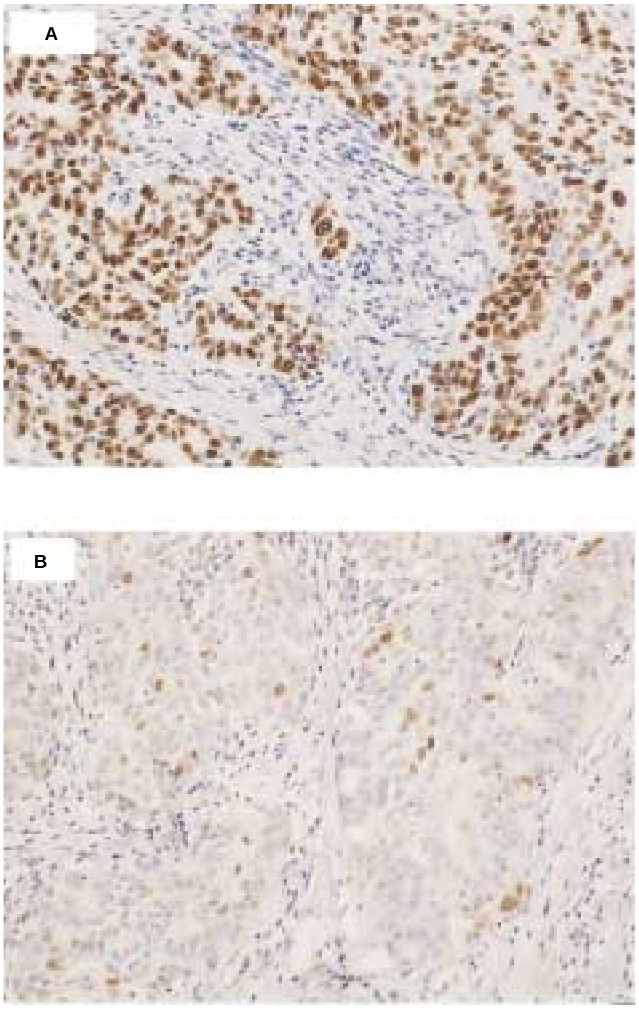

**Figure 3** Immunohistochemical staining for cancer number 8 showing (**A**) DO1 P53 staining, and (**B**) p21/WAF1 staining using Ab1

### DISCUSSION

FNA is clinically useful in the diagnosis of primary breast cancer and for the diagnosis and management of metastatic disease.

This study demonstrates that FNA can be used to assess protein expression in the p53 pathway using Western blotting. The technique thus provides a means to perform serial sampling over time in vivo and could be used to identify and monitor early tumour response (or failure to respond) at the biochemical level before clinical responses are evident during neo-adjuvant chemotherapy.

No difference was observed in p53, p21 or p27 expression (either in quality or quantity) between samples taken pre-operatively and post-operatively. This suggests that manipulation and potential short-term hypoxia during surgery does not affect p53 induction at the protein level, unlike human oesophageal cancer, where there is a marked effect on p53 induction by hypoxia (Hopwood et al, 1997). The additional species seen in the homogenised tumour (but not in the FNAs), is unlikely to be due to blood contamination. It could however be due to additional p53 species in the normal tissue given the greater proportion of cancer cells in an FNA than in a biopsy (Taxin et al, 1997), or due to early p53 protein degradation. In clinical practice, blood staining of FNAs is not uncommon; our data suggest that blood-stained FNA material is still suitable for p53 protein studies, although absolute protein concentration may not reflect that of the cancer cell population alone.

As expected, individual cancers showed different p53 levels within the panel probed with DO12 (Figure 2A) and with DO1 (data not shown) as we have previously described (Craig et al, 1999). This highlights the individuality of the cancers and their potentially different responses to treatment. DO1 recognises a site at the n-terminus of p53, which may be cleaved in some tumours, thus explaining why some samples were DO1 negative, but DO12 positive on Western blotting. Although DO1 is commonly used in histopathology to detect p53, DO12 is not amenable to IHC techniques. This emphasises the importance of using different antibodies (and techniques) when trying to unravel the p53 pathway in human breast cancer.

To further investigate the individuality of the p53 pathway in each tumour, the expression of p21/WAF1 and p27 (downstream targets of p53) was also examined by Western blotting and immunohistochemistry in the same set of tumours. These data confirm the complexity of the p53 pathway in breast cancer and suggest that there is another pathway involved in p21 activation possibly controlled by IRF-1 (Tanaka et al, 1996), as, unexpectedly, some samples were p53 negative, and p21 positive. Future in vivo studies could also study the mutational status of p53 in the cancer (Lavarino et al, 1998; Gudlaugsdottir et al, 2000), perhaps using the yeast functional assay (Flaman et al, 1995) which could be performed on FNA samples prior to chemotherapy.

The immunohistochemistry demonstrates concordance with detectable DO1, p21 and p27 expression from Western blots and confirmed that it was cancer cells rather than normal tissue expressing these proteins. However, the Western blots remain useful to identify which protein species predominate and could be used to identify and quantify changes between samples.

Based on this small series, FNA of breast cancers provides reproducible data on protein expression of p53, p21 and p27. FNA has the advantage over core biopsy or surgical biopsy, in that it is well tolerated by patients and can be performed on multiple occasions. Thus it could be used in vivo in the same patient to monitor changes in protein expression during treatment and hence predict and monitor the response to chemotherapy or radiotherapy of primary cancers or metastases in vivo.

© 2001 Cancer Research Campaign

### ACKNOWLEDGEMENTS

This work was funded by Breast Cancer Research (Scotland). We thank Gillian Scobie for technical assistance with the immunohistochemistry.

### REFERENCES

Aas T, Borresen AL, Geisler S, Smith-Sorensen B, Johnsen H, Varhaug JE, Akslen LA and Lonning PE (1996) Specific p53 gene mutations are associated with de novo resistance to doxorubicin in breast cancer patients. *Nat Med* **2**: 811–814

Andersen TI and Borrensen AL (1995) Alterations of the TP53 gene as a potential prognostic marker in breast carcinomas. Advantages of using constant denaturant gel electrophoresis in mutation detection. *Diagn Mol Path* **4**: 203–211

Barbareschi M, Caffo O, Doglioni C, Fina P, Marchetti A, Buttitta F, Leek R, Morelli L, Leonardi E, Bevilacqua G, Dalla Palma P and Harris AL (1996) p21WAF1 immunohistochemical expression in breast carcinoma: correlations with clinicopathological data, oestrogen receptor status, MIB1 expression, p53 gene and protein alterations and relapse-free survival. *Br J Cancer* **74**(2): 208–215

Blaydes JP and Hupp TR (1998) DNA damage triggers DRB-resistant phosphorylation of human p53 at the CK2 site. *Oncogene* **17**(8): 1045–1052

Blaydes JP, Craig AL, Wallace M, Ball HML, Traynor NJ, Gibbs NK and Hupp TR (2000) Synergistic activation of p53-dependent transcription by two cooperating damage recognition pathways. *Oncogene* **19**: 3829–3839

Bradford MM (1976) A rapid and sensitive method for the quantitation of microgram quantities of protein utilising the principle of protein dye binding. *Annal Biochem* **72**: 248–252

Brotherick I, Shenton BK and Lennard TW (1995) Are fine needle breast aspirated representative of the underlying solid tumour? *Br J Cancer* **72**: 732–737

Bueso-Ramos CE, Manshouri T, Haidar MA, Yang Y, McCown P, Ordonez N, Glassman A, Sneige N and Albitar M (1996) Abnormal expression of MDM-2 in breast carcinomas. *Breast Cancer Res Treat* **37**: 179–188

Craig Al, Blaydes JP, Burch LR, Thompson AM and Hupp TR (1999) Dephosphorylation of p53 at Ser20 after cellular exposure to low levels of non-ionizing radiation. *Oncogene* **18**: 6305–6312

Elledge RM and Allred DC (1998) Prognostic and predictive value of p53 and p21 in breast cancer. *Breast Cancer Res Treat* **52**: 79–98

Elledge RM, Lock-Lim S, Allred DC, Hilsenbeck SG and Cordner L (1995) p53 Mutation and Tamoxifen Resistance in Breast Cancer. *Clin Cancer Res* **1**: 1203–1208

Flaman JM, Frebourg T, Moreau V, Charbonnier F, Martin C, Chappuis P, Sappino AP, Limacher JM, Bron L, Benhattar J, Tada M, Van Meir EG, Estreicher A and Iggo RD (1995) A simple p53 functional assay for screening cell lines, blood and tumours. *Proc Natl Acad Sci* **92**: 3963–3967

Gudlaugsdottir S, Sigurdardottir V, Snorradottir M, Jonasson JG, Ogmundsdottir H and Eyfjord JE (2000) p53 mutation analysis in benign and malignant breast lesions: using needle rinses from fine-needle aspirations. *Diagn Cytopathol* **22**(5): 268–274

Hartley MN, Tuffnell DJ, Hutton JL, Palmer M and Al-Jafari MS (1988) Fine needle aspiration cytology: an in vitro study of cell yield. *Br J Surg* **75**(4): 380–381

Haupt Y, Maya R, Kazaz A and Oren M (1997) Mdm2 promotes the rapid degradation of p53. *Nature* **387**: 296–299

Hopwood D, Moitra S, Vojtesek B, Johnston DA, Dillon JF and Hupp TR (1997) Biochemical analysis of the stress protein response in human oesophageal epithelium. *Gut* **41**(2): 156–163

Jones JM, Cui XS, Medina D and Donehower LA (1999) Heterozygosity of p21/WAF1/CIP1 enhances tumour cell proliferation and cyclin D1-associated kinase activity in a murine mammary cancer model. *Cell Growth Differ* **10**: 213–222

Kastan MB, Onyekwere O, Sidransky D, Vogelstein B and Craig RW (1991) Participation of p53 protein in the cellular response to DNA damage. *Cancer Res* **51**: 6304–6311

Kuner R, Pollow K, Lehnert A, Pollow B, Scheler P, Krummenauer F, Casper F and Hoffmann G (2000) Needle biopsy vs. Conventional surgical biopsy – biochemical analysis of various prognostic factors. *Zentralbl Gynakol* **122**(3): 160–164

Lavarino C, Corletto V, Mezzelani A, Della Torre G, Bartoli C, Riva C, Pierotti MA, Rilke F and Pilotti S (1998) Detection of TP53 mutation, loss of heterozygosity and DNA content in fine needle aspirates of breast carcinoma. *Br J Cancer* **77**(1): 125–130

Linke SP, Clarkin KC, Di Leonardo A, Tsou A and Wahl GM (1996) A reversible p53-dependent GO/G1 cell cycle arrest induction by ribonucleotide depletion in the absence of detectable DNA damage. *Genes Dev* **10**: 934–947

Lowe SW, Bodics S, McClatchey A, Remington L, Ruley HE, Fisher DE, Housman DE and Jacks T (1994) p53 status and the efficacy of cancer therapy in vivo. *Science* **226**: 807–810

Makris A, Allred DC, Powles TJ, Dowsett M, Fernando IN, Trott PA, Ashley SE, Ormerod MG, Titley JC and Osborne CK (1997) Cytological evaluation of biological prognostic markers from primary breast carcinomas. *Breast Cancer Res Treat* **44**(1): 65–74

Nizzoli R, Bozzetti C, Naldi N, Guazzi A, Gabrielli M, Michiara M, Camisa R, Barilli A and Cocconi G (2000) Comparison of the results of immunocytochemical assays for biologic variables on preoperative fine-needle aspirates and on surgical specimens of primary breast carcinomas. *Cancer* **90**(1): 61–66

Pelosi G, Bresaola E, Rodella S, Manfrin E, Piubello Q, Schiavon I and Iannucci A (1994) Expression of proliferating cell nuclear antigen, Ki-67 antigen, oestrogen receptor protein and tumour suppressor p53 gene in cytological samples of breast cancer: an immunochemical study with clinical, pathobiological and histological correlations. *Diagn Cytopath* **11**(2): 131–140

Rao JY, Apple SK, Hemstreet GP, Jin Y and Nieberg RK (1998) Single cell multiple biomarker analysis in archival breast fine needle aspiration specimens: quantitative fluorescence image analysis of DNA content, p53 and G-actin as breast cancer biomarkers. *Cancer Epidemiological Biomarkers Prev* **11**: 1027–1033

Sgambato A, Zhang YJ, Arber N, Hibshoosh H, Doki Y, Ciaparrone ME, Santella RM, Cittadini A and Weinstein IB (1997) Deregulated expression of p27 (Kip1) in human breast cancers. *Clin Cancer Res* **3(**10**)**: 1879–1887

Sherr CJ and Roberts JM (1999) CDK inhibitors: positive and negative regulators of $G_1$-phase progression. *Genes Dev* **13**: 1501–1512

Shiohara M, Koike K, Komiyama A and Koeffler HP (1997) p21WAF1 mutations and human malignancies. *Leuk Lymphoma* **26**: 1–2, 35–41

Steele RJC, Thompson AM, Hall PA and Lane DP (1998) The p53 tumour suppressor gene. *Br J Surg* **85(**11**)**: 1460–1467

Stephen CW, Helminen P and Lane DP (1995) Characterisation of epitopes on human p53 using phage-displayed peptide libraries: insights into antibody - peptide interactions. *J Mol Biol* **248**(1): 58–78

Tanaka N, Ishihara M, Lamphier MS, Nozawa H, Matsuyama T, Mak TW, Aizawa S, Tokino T, Oren M and Taniguchi T (1996) Cooperation of the tumour suppressors IRF-1 and p53 in response to DNA damage. *Nature* **382**: 816–819

Taxin A, Tartter PI and Zappetti D (1997) Breast Cancer Diagnosis by Fine Needle Aspiration and Excisional Biopsy. Recurrence and Survival. *Acta Cytolgica* **41**(2): 302–306

Vojtesek B, Dolezalova H, Lauerova L, Svitakova M, Havlis P, Kovarik J, Midgley CA and Lane DP (1995) Conformational changes in p53 analysed using new antibodies to the core DNA binding domain of the protein. *Oncogene* **10**(2): 383–393

Ziyaie D, Hupp TR and Thompson AM (2000) p53 and breast cancer. *The Breast* **9**: 239–246

*© 2001 Cancer Research Campaign*
